# miR-573 inhibits prostate cancer metastasis by regulating epithelial-mesenchymal transition

**DOI:** 10.18632/oncotarget.5427

**Published:** 2015-10-03

**Authors:** Lin Wang, Guanhua Song, Weiwei Tan, Mei Qi, Lili Zhang, Jonathan Chan, Jindan Yu, Jinxiang Han, Bo Han

**Affiliations:** ^1^ Research Center for Medicinal Biotechnology, Key Laboratory for Rare & Uncommon Diseases of Shandong Province, Shandong Academy of Medicinal Sciences, Jinan, China; ^2^ Institute of Basic Medicine, Shandong Academy of Medical Sciences, Jinan, China; ^3^ Department of Pathology, Shandong University Medical School, Jinan, China; ^4^ Department of Immunology, University of Toronto, Toronto, Ontario, Canada; ^5^ Division of Hematology/Oncology, Department of Medicine, Northwestern University Feinberg School of Medicine, Chicago, IL, USA; ^6^ Department of Pathology, Shandong University Qilu Hospital, Jinan, China

**Keywords:** prostate cancer, miR-573, FGFR1, GATA3, metastasis

## Abstract

The metastastic cascade is a complex process that is regulated at multiple levels in prostate cancer (PCa). Recent evidence suggests that microRNAs (miRNAs) are involved in PCa metastasis and hold great promise as therapeutic targets. In this study, we found that miR-573 expression is significantly lower in metastatic tissues than matched primary PCa. Its downregulation is correlated with high Gleason score and cancer-related mortality of PCa patients (*P* = 0.041, Kaplan-Meier analysis). Through gain- and loss-of function experiments, we demonstrated that miR-573 inhibits PCa cell migration, invasion and TGF-β1-induced epithelial-mesenchymal transition (EMT) *in vitro* and lung metastasis *in vivo*. Mechanistically, miR573 directly targets the fibroblast growth factor receptor 1 (FGFR1) gene. Knockdown of FGFR1 phenocopies the effects of miR-573 expression on PCa cell invasion, whereas overexpression of FGFR1 partially attenuates the functions of miR-573. Consequently, miR-573 modulates the activation of FGFR1-downstream signaling in response to fibroblast growth factor 2 (FGF2). Importantly, we showed that GATA3 directly increases miR-573 expression, and thus down-regulates FGFR1 expression, EMT and invasion of PCa cells in a miR-573-dependent manner, supporting the involvement of GATA3, miR-573 and FGFR1 in controlling the EMT process during PCa metastasis. Altogether, our findings demonstrate a novel mechanism by which miR-573 modulates EMT and metastasis of PCa cells, and suggest miR-573 as a potential biomarker and/or therapeutic target for PCa management.

## INTRODUCTION

Metastasis is often responsible for the recurrence, poor prognosis and death of prostate cancer (PCa). Thus, a better understanding the molecular mechanisms involved in PCa metastasis will contribute to the identification of novel prognostic biomarkers and therapeutic targets [1]. MicroRNAs (miRNAs) are noncoding small RNAs that post-transcriptionally regulate protein expression [2]. Aberrant expression of many miRNAs has been linked to human diseases including cancers [2]. Like classical oncogenes or tumor suppressors, miRNAs have been shown to play important roles in cancer metastasis [3].

Epithelial-mesenchymal transition (EMT), characterized by the loss of epithelial characteristics and acquisition of a mesenchymal phenotype, plays a critical role in PCa progression and metastasis [4]. Reported molecular mechanisms underlying EMT involve regulation of transcription factors, cellular junctions, cytoskeleton, and miRNAs [4, 5]. For example, miR-154 and 379 were identified as oncogenes that promote EMT, whereas miR-30, 200, 145, 34a, 100, and 143 were found to act as tumor suppressor genes that inhibit this process [6–8]. Therefore, detailed research focusing on miRNAs might reveal novel mechanisms of PCa progression and potential targets for therapeutic intervention.

In this study, our data highlight the role of miR-573 in inhibiting PCa metastasis through targeting FGFR1 and therefore disrupting EMT process. In addition, miR-573 itself is a transcriptional target of GATA3, which is thus able to regulate FGFR1 expression and EMT in a miR-573-dependent manner. This GATA3-miR-573-FGFR1 axis may represent a central pathway that controls EMT during PCa progression and thus hold great promise for therapeutic intervention in the future.

## RESULTS

### miR-573 is down-regulated in metastatic PCa

**Figure 1:**
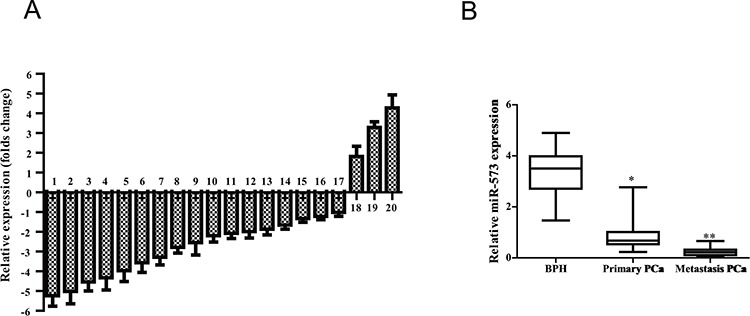
miR-573 level is decreased in human prostate cancer tissues **A.** Quantitation of 20 miRNAs were performed using qRT-PCR in three matched-pairs of primary and metastasis tissues and the results are shown as folds number in metastatic to primary ones. 1–20 represents miR-199, miR-573, miR-505, miR-449, miR-107, miR-23b, miR-22, miR-26b, miR-26a, miR-149, miR-455, miR-195, miR-331, miR-146a/b, miR-374b, miR-184, miR-301a, miR-92, miR-223 and miR-374a, respectively. **B.** qRT-PCR was further applied to investigate the miR-573 expression in BPH (*n* = 12), primary tumor (*n* = 65) and metastatic tissues (*n* = 15). *p < 0.05, **p < 0.01. Data are means of biological triplicates (± standard error).

To identify metastasis-related miRNAs, we comparatively analyzed the expression of 20 miRNAs in matched pairs of primary and metastatic tissues from three PCa patients. Of note, all of these miRNAs were previously reported to be highly dysregulated in PCa but their exact roles remained unclear [6–10]. Seven of the miRNAs, including miR-199, miR-573, miR-505, miR-449, miR-107, miR-23b and miR-22, were significantly repressed in metastasic PCa when compared with the matched primary tumors (folds change, Figure 1A). We selected miR-573 for further investigation as it was progressively down-regulated in BPH, primary and metastatic PCa tissues (Figure 1B) and its role in PCa was completely unknown.

### miR-573 suppresses PCa metastasis *in vitro* and *in vivo*

**Figure 2:**
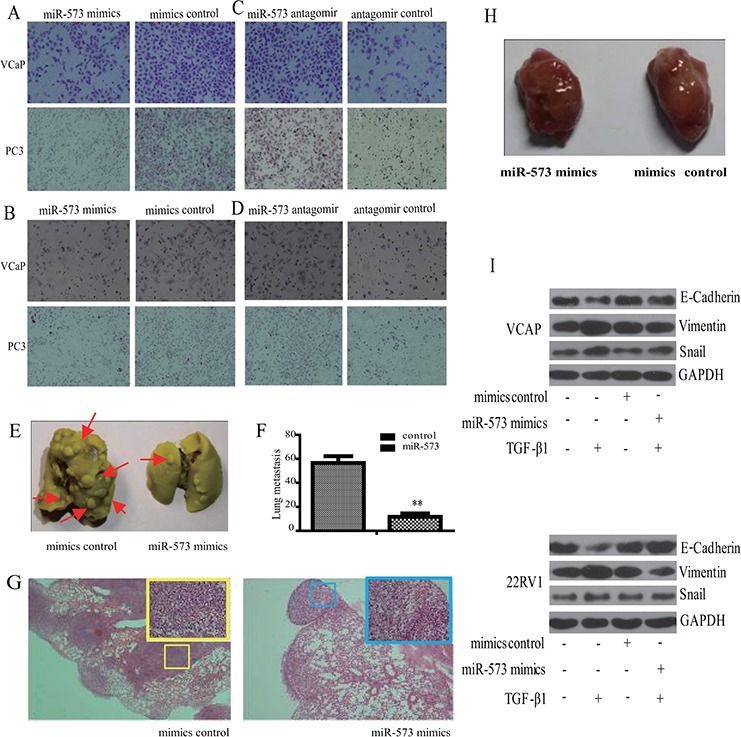
Effect of miR-573 on cell motility *in vitro* and and tumor metastasis *in vivo* Transwell migration **A, B.** and invasion **C, D.** assays were applied to detect the invasive and migratory potentials of VCaP and PC3 cells after transfection with the miR-573 mimics (left), its antagomir (right) or their controls, respectively (200 nM). **E.** VCaP stably transfected with miR573 and its controls were injected subcutaneously into the flanks of nude mice (*n* = 10). The metastatic foci in the lung is shown in (E) and quantified in **F. G.** Representative H&E staining images of lungs from one mouse per treatment group are shown. miR-573 mimics transfection showed significantly reduced number of metastatic lesions (red arrow). **H.** Tumor volumes of tumor mouse model were measured. **I.** Western blot was performed to detect the expression of E-cadherin, Vimentin and Snail in VCaP and 22RV1 cells during TGF-β1 induced EMT. Mimics/antagomir control: cells transfected with the mimics/antagomir control; miR-573 mimics/antagomir: cells transfected with the miR-573 mimics/antagomir. *p < 0.05, **p < 0.01. Data in F are means of biological triplicates (± standard error) and are representative of duplicate (E–H) or triplicate (A–D) experiments.

To understand the biological roles of miR-573 dysregulation in PCa cells, we performed *in vitro* gain- and loss-of-function analyses in VCaP and PC3 prostate cancer cell lines transfected with miR-573 mimics and antagomir, respectively. Matrigel-coated (for invasion) or -uncoated (for migration) transwell assays showed that ectopic miR-573 expression significantly inhibited VCaP and PC3 cell migration (Figure 2A) and invasion (Figure 2B). By contrast, miR-573 antagomir treatment led to markedly increased PCa cell invasion and migration (Figure 2C and Figure 2D).

We next tested whether miR-573 could inhibit metastasis *in vivo* using a retroviral overexpression strategy. VCaP cells transfected with miR-573 or vector control were injected into the dorsal flank of nude mice (*n* = 10). Notably, mice transplanted with control VCaP cells displayed prominent lung metastasis (*n* = 56.5 ± 5.7), whereas mice bearing VCaP/miR-573 mimics tumors showed significantly less metastasis (*n* = 11.6 ± 2.9, *p* = 0.0037 < 0.01) (Figure 2E and Figure 2F). The corresponding HE images of the lungs are shown in Figure 2G. By contrast, the weight of the primary tumors from miR-573 mimics (935.6 ± 59.8 mm^3^) and control mimics (962.6 ± 72.8 mm^3^) mice were hardly affected (Figure 2H), which were consistent with our findings by MTS assays *in vitro* (data not shown). Collectively, our data suggest that miR-573 significantly suppresses PCa invasion and metastasis.

### miR-573 inhibits the invasive capacity of PCa cells by regulating EMT process

As EMT is an important cellular process that augments cell motility and leads to tumor metastasis [11], we wondered whether miR-573 is involved in EMT of PCa cells. TGF-β1 has emerged as a potent inducer of EMT, as well as a factor for the maintenance of EMT in a variety of cancers [12]. As shown in Figure 2I, TGF-β1 treatment in VCaP and 22RV1 cells led to a significant inhibition of epithelial marker (E-cadherin), but induction of mesenchymal markers (Vimentin and Snail). Importantly, overexpression of miR-573 restored E-cadherin expression while blocked the induction of vimentin and Snail, supporting an EMT-inhibitory role.

### miR-573 targets metastasis regulator FGFR1

**Figure 3:**
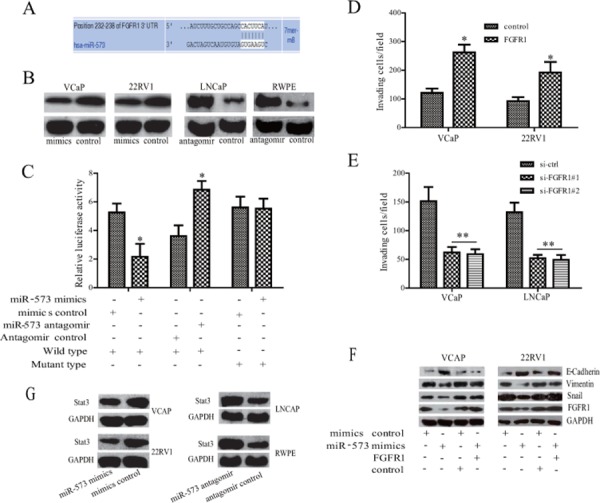
FGFR1 is directly targeted by miR-573 during EMT process **A.** The 3′-UTR element of EGFR1 messenger RNA is partially complementary to miR-573. **B.** Western blot was performed to detect the expression of FGFR1 in different kinds of cells after the indicated transfection. **C.** Luciferase reporter assay was used to analyse whether FGFR1 was a direct target of miR-573. HEK293T cells were co-transfected with FGFR1–3′-UTR luciferase reporter, wild or mutant-type, mimicss control or miR-573 mimicss and antagomir control or miR-573 antagomir for 48 hours before analysis. Firefly luciferase activity of the reporter was normalized to the internal Renilla luciferase activity. The means ± SD of three independent experiments are shown. **D.** VCaP and 22RV1 cells were co-transfected with FGFR1 or empty vector together with miR-573. Forty-eight hours later, transwell assay was applied. **E.** VCaP and LNCaP were transfected with si-ctrl or with si-FGFR1 together with miR-573 antagomir. Forty-eight hours later, invasion ability was detected by Transwell apparatus. **F.** E-cadherin, Vimentin and Snail were assayed by Western blot analysis. **G.** The Stat3 expression was detected by Western blot analysis in various PCa cell lines after the indicated transfection. *p < 0.05, **p < 0.01. Data in D and E are means of biological triplicates (± standard error) and all data are representative of duplicate experiments.

To understand the molecular mechanism by which miR-573 inhibits PCa EMT, invasion and metastasis, we analyzed putative targets of miR-573 using two bioinformatics tools, TargetScan [13] and miRWalk [14], and identified a set of 10 genes that are most likely to be targeted by miR-573. From this set, we further selected the ones with relevance to PCa based on their associated Gene Ontology (GO) terms [15]. The most notable target gene was FGFR1 (Figure 3A), which is known to play a critical role in prostate tumorigenesis and particularly in EMT and metastasis [16]. Western blot experiments confirmed a clear inhibition of FGFR1 upon transfection with miR-573 mimics in VCaP and 22RV1 cells, but an obvious increase upon miR-573 knockdown in LNCaP and RWPE cells (Figure 3B). Furthermore, reporter assays showed that the luciferase activity of the 3′ UTR of FGFR1 gene was significantly reduced in miR-573 mimics–transfected HEK293T cells, but markedly increased by miR-573 antagomir treatment (Figure 3C). To further demonstrate a direct interaction of miR-573 with the FGFR1 3′ UTR, site-directed mutagenesis of the miR-573 binding sites was performed. As shown in Figure 3C, these mutations completely abolished the repressive effects of miR-573 on FGFR-1 3′ UTR activities. Collectively, our data suggests that miR-573 directly suppresses FGFR1 expression in PCa cells.

Next, we investigated the effects of FGFR1 on the invasive capacity in miR-573–transduced VCaP and 22RV1 cells. As shown in Figure 3D, ectopically expressing FGFR1 in VCaP and 22RV1 cells restored miR-573 mimics–impaired invasiveness. On the contrary, FGFR1 silencing in miR-573 antagomir-treated VCaP and LNCaP cells decreased cell invasion (Figure 3E). Further study demonstrated that overexpression of FGFR1 in VCaP and 22RV1 cells could attenuated the inhibitory effects of miR-573 on EMT as indicated by the downregulation of E-cadherin and upregulation of Vimentin and Snail (Figure 3F). As Stat3 is an important target in FGFR1 signaling [17, 18], we next demonstrated that Stat3 expression is significantly decreased in miR-573 mimics-transfected VCaP and 22RV1 cells, but increased after its antagomir transfection in LNCaP and RWPE cells (Figure 3G). Altogether, our results indicate that FGFR1 is an important mediator for the function of miR-573 in PCa cells.

### miR-573 inhibits FGFR1-downstream signaling pathways

**Figure 4:**
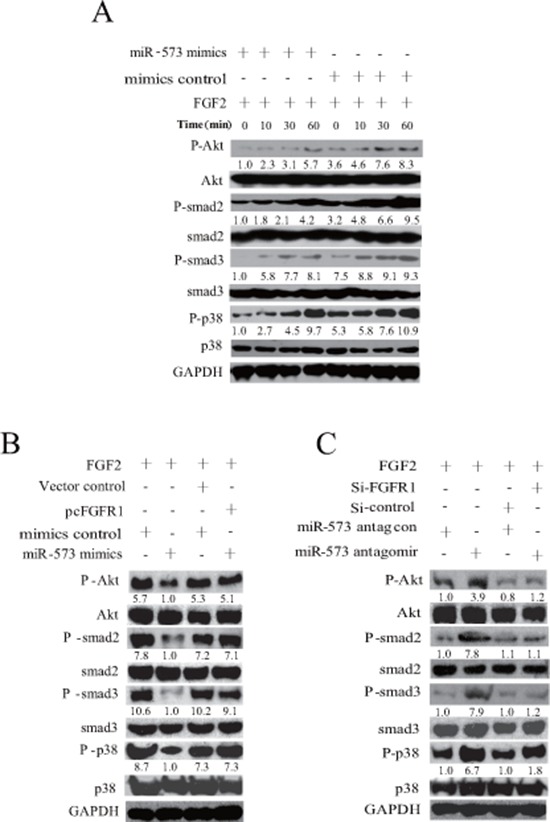
miR-573 inhibits FGFR1 signaling **A.** Phospgorylation and total levels of Akt, smad2, smad3, p38 was assayed by Western blot with the FGF2 stimulation in VCaP cells after miR-573 mimicss or its control transfection. **B.** After co-transfection with lentivirus of miR573 and FGFR1 expression plasmids in VCaP cells, activation of signaling pathways was measured by Western blot in response to FGF2. **C.** Effects of silencing FGFR1 on Akt, smad2, smad3, p38 activation in VCaP cells with/without miR-573 antagomir transfection by Western blotting. The quantification of each protein band in the result of Western blotting was done using LAS-3000 with MultiGauge software (Fuji film). The amount of phosphorylated protein was normalized versus that of total ones. Data are representative of triplicate experiments.

In light of the critical role of FGFR1 in tumorigenesis and metastasis [19], especially in mediating the activation of PI3K/Akt, Smad2/3 and p38 pathways [20], we asked whether miR-573-triggered suppression of FGFR1 regulates these signaling pathways in VCaP cells. Strikingly, overexpression of miR-573 attenuated the activity of AKT, Smad2, Smad3, p38 signaling after FGF2 stimulation at various time points (Figure 4A). Later, we analyzed the activity of the above signaling after overexpressing FGFR1 in miR-573 mimics-transduced VCaP cells. As shown in Figure 4B, overexpression of FGFR1 blocked the inhibitory effects of miR-573 mimics on FGFR1 signaling (Akt, Smad2, Smad3 and p38), whereas knockdown of FGFR1 recapitulated the effect of miR-573 antagomir on their activation in response to FGF2 stimulation under serum starvation conditions (Figure 4C). These results corroborated the adverse correlation between miR-573 and FGFR1 regulation in PCa.

### GATA–3 induces miR-573 expression in PCa

**Figure 5:**
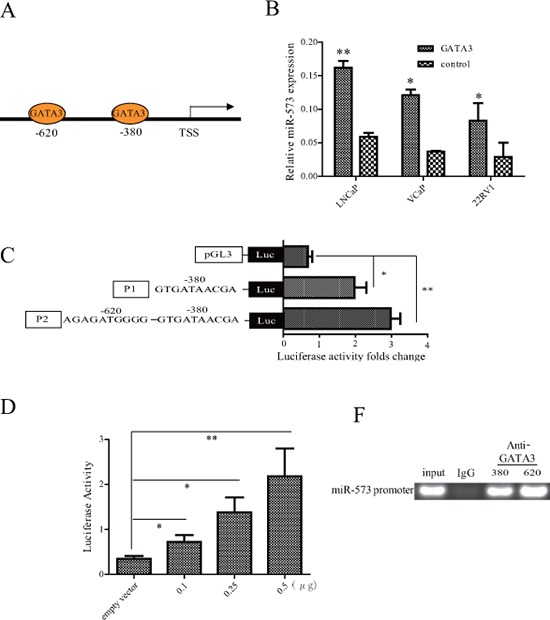
The expression of miR-573 was directly regulated by GATA3 **A.** The putative GATA3 binding sites in two gene promoter regions. **B.** Expression of miR-573 was determined by qRT-PCR after overexpressing GATA3 in indicated cell lines. **C.** Luciferase activity was assayed after co-transfection with GATA3 expression plasmid and miR57-pGL3-pro constructs with different lengthens of its promoter. **D.** Luciferase assay of miR-573 transcriptional activity in HEK392T cells after transfection with GATA3 expression plasmid of increased concentrations. **E.** ChIP was performed using an anti-GATA3 antibody and a rabbit IgG as the control. *p < 0.05, **p < 0.01. Data in (B–D) are means of biological triplicates (± standard error) and are representative of triplicate (B–E) experiments.

In an attempt to address how miR-573 became downregulated in PCa, we hypothesized that an unknown transcriptional factor might play a role in the regulation of miR-573. The miRBase Sequences database was firstly utilized to identify the human mature miR-573 sequence. Sequence analysis with TESS and TFSEARCH databases was subsequently performed and we identified two conserved GATA3 motifs in the miR-573 promoter (Figure 5A). GATA3 has been reported to play a critical role in the development of several tissues [21, 22] and its inactivation promotes tumor progression in PCa through regulating miRNA, such as miR-29 [23]. To determine how GATA3 regulates miR-573 in PCa, we overexpressed GATA3 (Figure 5B) in LNCaP, VCaP and 22RV1, and found a significant increase in miR-573 levels. Next, we co-transfected HEK293T cells with miR-573 promoter fragments of different lengths with either GATA3-vector or vector control and assessed the relative luciferase activity 48 h after transfection. The results revealed that co-transfection with GATA3-vector upregulated the luciferase activity of the 620bp and 380bp fragments but not the 300 bp fragment (Figure 5C), which suggests that the GATA3 binding sites drive GATA3-induced luciferase activity in PCa cells. Further, the luciferase activity of HEK293T cells transfected with the 620bp fragment increased steadily with increasing GATA3 concentration (Figure 5D), suggesting that the luciferase activity of these 2 sites are specific and driven by GATA3. ChIP analysis further corroborated that GATA3 binds to the promoter of miR-573 (Figure 5E).

### miR-573 mediates the ability of GATA3 in suppressing PCa metastasis

**Figure 6:**
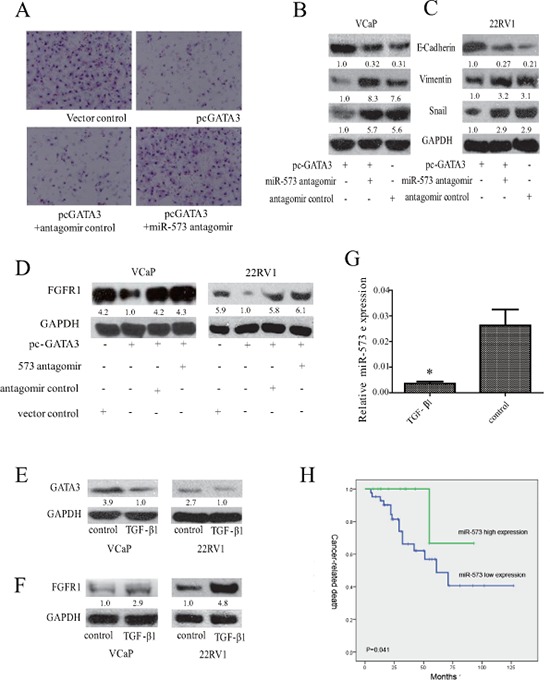
Association of miR-573 expression with FGFR1 and GATA3 in PCa **A.** The invasive ability was measured by Transwell apparatus after the indicated transfeciton. Expression of E-cadherin, Vimentin and Snail were examined by Western blot after co-transfection with GATA3 vector or its control, and miR-573 antagomir or its control in VCaP **B.** and 22RV1 **C. D.** Western blot was applied to assay the expression of FGFR1 in VCaP and 22RV1 after indicated transfection. The expression levels of GATA3 **E.** and FGFR1 **F.** were detected by Western blot in VCaP and 22RV1 during TGF-β1 treament. **G.** miR-573 expression was detected in VCaP and 22RV1 cells after TGF-beta1 treatment by qRT-PCR. **H.** Kaplan-Meier metastasis-free survival curve comparing 2 groups of high (greater than the mean; *n* = 22) and low (less than the mean; *n* = 43) miR-573 expression level in the tumors of 65 localized PCa patients; A total of 55 PCa patients were available for prognostic analysis. *P* value is based on a log-rank test. *p < 0.05. Data C is means of biological triplicates (±standard error) and are representative of triplicate (A–E) experiments. The quantification of protein bands in Western blot was done using LAS-3000 with MultiGauge software.

To determine whether GATA3-mediated suppression of PCa metastasis involves miR-573, we transfected VCaP-GATA3 cells with miR-573 antagomir. Our data showed that GATA3 overexpression inhibited invasion of PCa cells, but concomitant inhibition of miR-573 abrogated these effects of GATA3 (Figure 6A). In addition, consistent with a previous study on breast cancer [24], ectopic expression of GATA3 could also inhibit TGF-β1-induced EMT in 22RV1, as evident by increased E-cadherin expression and decreased Vimentin and Snail expression (Supplementary Figure S1). To determine whether miR-573 is essential to GATA3-mediated inhibition of EMT, we transfected miR-573 antagomir into VCaP (Figure 6B) and 22RV1 (Figure 6C) cells. As expected, miR-573 antagomir abrogated the effects of GATA3 overexpression as demonstrated by the increase in the expression levels of epithelial markers (E-cadherin, Figure 6B and 6C) and decrease of mesenchymal ones (Vimentin and Snail, Figure 6B and 6C). Thus, tumor cells acquire mesenchymal characteristics in the absence of miR-573, even with high levels of GATA3 expression. Further studies demonstrated that overexpressed GATA3 in VCaP and 22RV1 cells could down-regulate FGFR1 expression, whereas simultaneous miR-573 antagomir transfection attenuated this effect, suggesting that miR-573 is an important mediator of GATA3 that controls the expression of pro-metastatic genes, ultimately leading to metastasis suppression (Figure 6D).

We also characterized the expression of GATA3, miR-573 and FGFR1 during TGF-β1-induced EMT in VCaP and 22RV1 cells. After TGF-β treatment for 72 h, the expression of GATA3 was significantly downregulated (Figure 6E), but FGFR1 increased instead (Figure 6F). As expected, the expression level of miR-573 significantly decreased accordingly (Figure 6G). These results further support GATA3, miR-573 and FGFR1 as cooperative mediators of TGF-β1 induced EMT of PCa.

### Clinical significance of miR-573 expression in human PCa

To further understand the clinical relevance of the above findings in human PCa, the expression of miR-573 was examined in 65 PCa cases. As shown in Table 1, non-parametric test revealed that low miR-573 level was significantly associated with higher Gleason Score (*p* = 0.044), but not with age, PSA level, or tumor grade.

**Table 1:** Correlations between miR-573 expression and clinicopathological characteristics in prostate cancer patients

Parameters	N (cases)	miR-573 expression (%)		*P* value
		Low	High	
Age(year)				
<55	18	11(61.1)	7(38.9)	0.714
≥55	47	32(68.1)	15(31.9)	
Pre PSA(ng / ml)				
<4	6	4(66.7)	2(33.3)	0.801
4–10	7	4(57.1)	3(42.9)	
>10	52	35(67.3)	17(32.7)	
Gleason score				
<7	16	6(37.5)	10(62.5)	0.044
7	23	10(43.5)	13(56.5)	
>7	26	18(69.2)	8(30.8)	
Clinical tumor stage				
≤cT2	48	30(62.5)	18(37.5)	0.776
≥cT3	17	13(76.5)	4(23.5)	
Ki67				
<10%	52	35(67.3)	17(32.7)	0.694
≥10%	13	8(61.5)	5(38.5)	
FGFR1				
Not overexpressed	43	24(55.8)	19(44.2)	0.029
Overexpressed	22	19(86.4)	3(13.6)	
GATA-3				
Not expressed	60	42(70.0)	18(30.0)	0.075
Expressed	5	1(20.0)	4(80.0)	

Next, we compared cancer-related survival rates between patients with high or low miR-573 levels in univariate models. Kaplan-Meier survival analysis showed that the group of patients who had higher miR-573 levels had a less rate of mortality (*p* = 0.041 Figure 6H).

We next determined whether there was an association between the expression levels of miR-573 and its targets in clinical PCa tissues. Significantly, as shown in Table 1, miR-573 levels were inversely correlated with the expression of FGFR1 (*p* = 0.029). Accordingly, there was a tendency for association between GATA3 expression and high miR-573 level in PCa cases, although it did not reach a significant difference (*p* = 0.075).

## DISCUSSION

Accumulating evidence suggests a causal role of miRNAs in the metastasis of human PCa due to their capacity in coordinately suppressing multiple target genes [24]. Previously, miR-573 has been reported to act as a tumor suppressor gene in melanoma, gastric and breast cancer [25]. In the current study, for the first time, we demonstrated that miR-573 exhibits a stepwise downregulation in BPH, primary PCa tumors and metastatic tissues. We also found that decreased level of miR-573 correlates with clinically relevant parameters, such as Gleason scores. More importantly, miR-573 repression coincides with gain of metastatic properties and is associated with poor prognosis. In all, these data suppport a potential involvement of miR-573 during the progression of PCa. With gain- and loss- of function experiments, we demonstrated that miR-573 inhibited the invasive capacity of PCa, but not cellular proliferation. Accordingly, over-expressing of miR-573 in tumor cells inhibited TGFβ1-induced EMT. Therefore these results clearly demonstrated that restoration of miR-573 might reverse EMT phenotypes and suppress metastasis of PCa cells.

Further, our study identified FGFR1 as a key downstream target of miR-573. As previously reported, FGFR1 is highly expressed in various malignancies and its overexpression induces a series of cellular and molecular changes associated with EMT in bladder cancer and PCa [16, 17]. Our present study demonstrated that upregulating FGFR1 could reverse miR-573-induced invasion and EMT inhibition, verifying that the EMT induced by miR-573 deficiency in PCa requires FGFR1 participation. Specifically, Stat3, which was related with PCa progression and EMT [26], was also identified to be regulated by miR-573, indicating that the mechanism of its involvement in PCa may be multiplex. Furthermore, it has been reported that, after binding with its ligands, FGFR could activate its downstream pathway, such as PI3K/Akt, p38 and smad2/3, and consequently impel the proliferation and invasion of tumor cells [19]. Our data showed that introduction of exogenous miR-573 into PCa cells could attenuate the activation of PI3K/Akt, p38 and smad2/3 after FGF2 treatment in a FGFR1-dependent way. Recently, targeting FGFR1 has been under development for clinical use in the treatment of cancer [27] and the FGFR1 inhibitor PD173074 induces mesenchymal-epithelial transition in head and neck squamous cell carcinoma [18]. These results suggest potent therapeutic value of miR-573 in PCa due to its role in targeting FGFR1 pathway.

Further, our data showed that miR-573 is a potential downstream effector of GATA3, which has been shown to be a key factor in preventing PCa progression in the castration-resistant PTEN-deficient model, through antagonizing Akt signalling [28]. GATA3 was found to physically bind to the responsive elements located upstream of miR-573 promoter, subsequently exerting a direct influence on its transcription. This is in line with previous report suggesting that decreased level of GATA3 correlates with higher E-cadherin and its overexpression inhibits metastasis in breast cancer [22, 29]. Importantly, GATA3 could suppress metastasis through modifying the tumor microenvironment, such as angiogenesis and macrophage infiltrations, indicating that miR-573, the target of GATA3, may also be involved in such microenvironmental signaling. Indeed, miR-573 could inhibit angiongenesis through regulating the production or activity of VEGF in breast cancer [29]. All these data further support that miR-573 plays anti-metastasis roles by regulating diverse cellular processes. On the other hand, GATA3 could also regulate FGFR1 expression in a miR-573-dependent manner, indicating that miR-573 is indeed functional in the regulation of PCa progression. In addition, our data showed that the expression of GATA3 and miR-573 decreased, whereas FGFR1 increased during TGF-β1-induced EMT. The existence of GATA3/miR-573/FGFR1 axis was further confirmed by our findings that miR-573 was negatively correlated with FGFR1 expression, and tends to be positively correlated with GATA3 expression in human PCa tissues. This supports the concept that GATA3 and miR-573 act collaboratively through targeting FGFR1 during the progression of PCa and their collaboration highlights its pathological significance.

In summary, our study demonstrated prostate tumorigenesis caused by miR-573 down-regulation, which links FGFR1-mediated oncogenic signaling pathways with GATA3 dysregulation in PCa. Therefore, miR-573 might be a potential biomarker and targeting this novel GATA3/miR-573/FGFR1 axis will be a promising strategy for the treatment of a subset of metastatic PCa.

## MATERIALS AND METHODS

### Tumor samples collection

Benign Prostate Hyperplasia (BPH, *n* = 12), primary localized PCa (*n* = 65) and metastatic tissues (*n* = 15) were collected and embedded in paraffin for histological diagnosis and immunohistolocical study. None of the localized PCa patients received preoperative radiation or androgen deprivation therapy. Of note, matched pairs of primary and metastatic tissues were obtained from three PCa patients. The tissue morphology was validated by two pathologists (B.H. and W.T.). Detailed clinical and pathological profile were obtained from medical records and maintained on a secure relational database. This study was approved by the Institutional Review Board at the School of Medicine of Shandong University. Clinicopathological characters of localized PCa patients included in this study are included in Table 1.

### Bioinformatic sequence analysis

The mature human miR-573 sequence was retrieved from the miRBase Sequence Database (http://microrna.sanger.ac.uk/sequences/, University of Manchester, UK). The sequence of the miR-573 promoter was retrieved from the ISB lab (http://mirstart.mbc.nctu.edu.tw, National Chiao Tung University, Hsinchu, Taiwan). Transcriptional factor binding sites were predicted by the Transcription Element Search System (http://www.cbil.upenn.edu/cgi-bin/tess/tess, University of Pennsylvania, PA, USA) and the TFSEARCH system (http://www.cbrc.jp/research/db/TFSEARCH.html, Kyoto University, Japan).

### Cell lines and reagents

Human Vcap, PC3, LNCaP, 22RV1, RWPE and embryonic kidney 293 (HEK293T) cells were obtained from the American Type Culture Collection (Rockville, MD, USA) and cultured by following the manufacturer's instructions. Recombinant human FGF basic (FGF2) was purchased from R&D Systems (Minneapolis, MN). 5-Aza-2′-deoxycytidine (5-Aza-dC) and Trichostatin A (TSA) were obtained from Sigma-Aldrich (Saint Louis, MO).

### SiRNA transfection and plasmids

Indicated siRNA, vectors and miR-573 mimics/antagomir transfections were carried out using Lipofectamine 2000 according to the manufacturer's protocol and each sequence is listed in Supplementary Table 1. Non-specific negative control siRNAs were also used (sense strand: 5′-UUCUCCGAACGUGUCACG-3′; anti-sense strand: 5′-ACGUGACACGUUCGGAGAATT-3′). The mock group was defined as the one supplemented with the transfection reagent only.

The wild-type and mutants FGFR1 3′-UTR were generated on the miR-573 target recognition sites (seed sequences) as shown in Supplementary Table S1. Both the wild-type and mutated 3′-UTRs of FGFR1 gene were cloned into the pmirGLO dual luciferase reporter vector using Sac I and Xba I restriction sites. Furthermore, a GATA3 expression vector was generated into a pCMV-N-flag vector (Beyotime, Jiangsu, China) using ECoR I and Xba I restriction sites. A 2,000-bp fragment of the miR-573 promoter region was PCR amplified from the genomic DNA of HEK293T cell line using primers in Supplementary Table S2. The PCR product was cloned into a pGL3 luciferase reporter (Promega Corp, Madison, WI, USA) and the resulting vector was named miR573-pGL3-pro. FGFR1 expression vector was bought from Addgene company and all these vector products were validated by sequencing.

### RNA extraction and real time RT-PCR (qRT-PCR)

Paraffin sections 10 mm thick were cut, dewaxed, rehydrated and lightly stained with hematoxylin. Tumor tissues were examined and microdissected under a dissecting microscope (Leica ASLMD, Witts Baden, Germany). miRNA extraction was performed with a miRNeasy FFPE kit (Qiagen, Hilden, Germany), which enabled copurification of total RNA, including miRNA, from formalin-fixed paraffin-embedded tissue sections. Expression of miR-573 was measured using SYBR Primescript miRNA RT-PCR kit and SYBR^®^ Green I (TOYOBO, Osaka, Japan) according to the manufacturer's instructions. Melting analysis of the PCR products was conducted to validate the amplification of the specific product. The universal small nuclear RNA U6 was used as an endogenous control for miRNAs.

### Immunohistochemistry (IHC)

IHC was performed as previously described [30]. Immunohistochemical staining was performed using the standardized labeled streptavidin biotin (LSAB) kit (Dako Cytomation, Carpinteria, CA) according to the manufacturer's instructions. The slides were incubated with rabbit polyclonal anti-GATA3 antibody (1:100 dilution, Abcam, Cambridge, MA, UK), and anti-FGFR1 anti-body (1:100 dilution, Abcam, Cambridge, MA, UK). The slides were evaluated blindly by two independent observers (B.H. And W.T.), and the scoring systems to validate FGFR1, GATA3 expression were described before [30].

### Migration and invasion assay

After transfection with the indicated miRNA, siRNA or plasmids, migration and invasion assays were performed as previously described [31].

### miRNA target predictions, luciferase activity assay and western blotting

Predicted targets for miRNAs and their target sites were analyzed using TargetScan [13], and miRwalk [14]. For reporter assays, HEK293T cells were transiently transfected with reporter constructs together with miRNA mimics/antagomir and mimicss/antagomir control. To assay miR-573 promoter activity, HEK293T was transfected with GATA3 expression plasmid, reporter construct and the TK-Renilla luciferase plasmid that was used as a transfection control. Cell extracts were prepared 30 h after transfection, and the ratio of Renilla to firefly luciferase was measured with the Dual-Luciferase Reporter Assay System (Promega Corp, Madison, WI, USA). Anti-FGFR1, anti-Stat3, anti-E-cadherin, anti-ZO1, anti-Vimentin, anti-Snail, anti-ZEB1 and anti-GAPDH (Cell signaling, Danvers, MA, USA) rabbit polyclonal antibodies were used in Western blotting and the protocols was described previously [32].

### Chromatin imunoprecipitation (ChIP) assay

Details were described previously [12] and the purified chromatin was immunoprecipitated using 2 μg of anti-GATA3 and irrelevant antibody (anti-IgG, Santa Cruz, Dallas, Texas, USA). The primers used were described in Supplementary Table S2.

### Mouse models of tumor growth and metastasis

Male Balb/c athymic nude mice at 6 weeks of age were carried out in strict accordance with the recommendations in the Guide for the Care and Use of Laboratory Animals of the National Institutes of Health. The protocol was approved by the Scientific Investigation Board of Medical School of Shandong University.

For measurement of tumor invasion and metastasis, 10 of these mice were injected subcutaneously with VCaP cells infected with adenoviruses expressing miR-573-EGFP and 10 mice were injected with VCaP cells infected with adenoviruses expressing control-EGFP (4 × 10^6^ cells per mouse). Metastatic lesions in the lungs were counted macroscopically 12–14 weeks after injection.

### Statistical analysis

Statistical analyses were carried out using the Statistical Package for Social Sciences, version 19.0 (SPSS, IBM crop, Armonk, NY, USA). Two-sided Student's *t*-test and Mann–Whitney test were used for statistical comparisons; correlations between miR-573 expression with clinicopathological parameters were evaluated by the Spearman's test. The Kaplan–Meier method tests were utilized for the analysis of follow-up data, and hazard ratio (HR) with 95% confidence intervals (CI) were calculated. A *P*-value of 0.05 was considered significant.

## SUPPLEMENTARY FIGURE AND TABLES


