# Supplementary material for: miR-573 inhibits prostate cancer metastasis by regulating epithelial-mesenchymal transition
Source: Oncotarget. 2015 Oct 3;6(34):35978–90. doi: 10.18632/oncotarget.5427 (PMC4742155; doi:10.18632/oncotarget.5427)
Supplement: Supplementary file 1 [file oncotarget-06-35978-s001.pdf]

SUPPLEMENTARY FIGURE AND TABLES

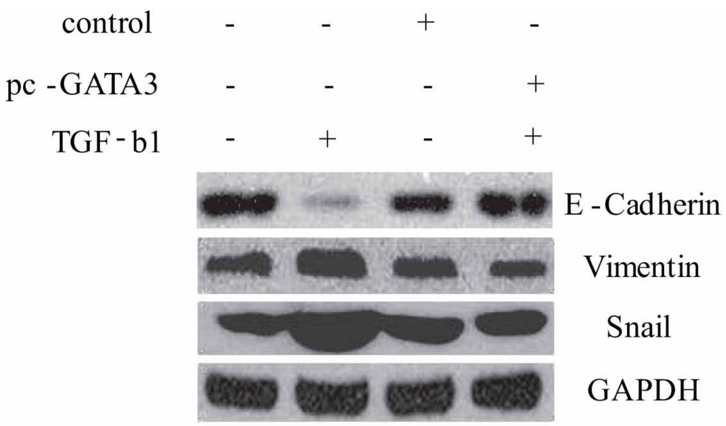

Supplementary Figure S1: GATA3 inhibits TGF-β1-induced EMT in PCa cells. The expression levels of E-Cadherin, Vimentin and Snail were detected by Western blotting after indicated treatments.

Supplementary Table S1: Details of siRNA used in this study

MicroRNA	Sequence	Catalog no.
573 mimic	CUGAAGUGAUGUGUAACUGAUCAG	SI02664431
573 inhibitor	CUGAAGUGAUGUGUAACUGAUCAG	SI02664053
siRNA		
FGFR1#1	AACAGATAACACCAAACCAAA	SI02757265
FGFR1#2	CAGAGATTTACCCATCGGGTA	SI02757258
GATA3#1	ACAGACCGAACTGTTGTATAA	SI00132433
GATA3#2	CACCGAAAGCAAATCATTCAA	SI00132447

Supplementary Table S2: Primers used in this study

Gene	Forward	Reverse
Vector		
GATA3	CGGAATTCCCATGGAGGTGACGGCG	GCTCTAGAGCCTAACCCATGGCGGT
TXNDC5	CGCGGATCCATGGAAGATGCCAAAGT	CCGCTCGAGCTAAAGTTCGTCTTTCG
FGFR1 3′UTR (wild type)	AAAAGTACTTTTAGAAAAAGAAGATGTC	CCCCCGGGGGCTGGTCACATGGATA
FGFR1 3′UTR (mutant type)	CTTTGCTGCCAGCCAGAAGATCCCCTCCCAGATG	CATCTGGGAGGGGATCTTCTGGCTGGCAGCAAAG
pGL3-basic-miR573(+295~−87)	CGAGCTCGTGTAAGATATTTACTT	CCCTCGAGGGAATTTGCATCTCAAA
pGL3-basic-miR573(+497~−87)	CGAGCTCGAGCTGGTATTGAGTTCT	CCCTCGAGGGAATTTGCATCTCAAA
pGL3-basic-miR573(+707~−87)	CGAGCTCGTCACAGGTTCAAGCGA	CCCTCGAGGGAATTTGCATCTCAAA
CHIP		
miR573-pro-380	TCACAGGTTCAAGCGA	AGAACTCAATACCAGCTG
miR573-pro-620	AGCTGGTATTGAGTTCTTA	AAGTAAATATCTTACAA
